# Lessons learned from unsolicited findings in clinical exome sequencing of 16,482 individuals

**DOI:** 10.1038/s41431-021-00964-0

**Published:** 2021-10-25

**Authors:** Vyne van der Schoot, Lonneke Haer-Wigman, Ilse Feenstra, Femke Tammer, Anke J. M. Oerlemans, Martine P. A. van Koolwijk, Frans van Agt, Yvonne H. J. M. Arens, Han G. Brunner, Lisenka E. L. M. Vissers, Helger G. Yntema

**Affiliations:** 1 Department of Clinical Genetics, Maastricht University Medical Center, PO Box 5800, 6202 AZ Maastricht, the Netherlands; 2 Department of Human Genetics, Radboud University Medical Center, PO Box 9101, 6500 HB Nijmegen, the Netherlands; 3 Donders Institute for Brain, Cognition and Behaviour, Radboud University Medical Center, Nijmegen, the Netherlands; 4 IQ Healthcare, Radboud Institute for Health Sciences, Radboud University Medical Center, PO Box 9101, 6500 HB Nijmegen, the Netherlands; 5 Commissie Mensgebonden Onderzoek, Research Ethics Committee, PO Box 9101, 6500 HB, Nijmegen, the Netherlands; 6 Radboud Institute for Molecular Life Sciences, Radboud University, Box 9101, 6500 HB Nijmegen, the Netherlands

**Keywords:** Health policy, Medical genetics

## Abstract

Unsolicited findings (UFs) are uncovered unintentionally and predispose to a disease unrelated to the clinical question. The frequency and nature of UFs uncovered in clinical practice remain largely unexplored. We here evaluated UFs identified during a 5-year period in which 16,482 index patients received clinical whole-exome sequencing (WES). UFs were identified in 0.58% (95/16,482) of index patients, indicating that the overall frequency of UFs in clinical WES is low. Fewer UFs were identified using restricted disease-gene panels (0.03%) than when using whole-exome/Mendeliome analysis (1.03%). The UF was disclosed to 86 of 95 individuals, for reasons of medical actionability. Only 61% of these UFs reside in a gene that is listed on the “ACMG59”-list, representing a list of 59 genes for which the American College of Medical Genetics recommends UF disclosure. The remaining 39% were grouped into four categories: disorders similar to “ACMG59”-listed disorders (25%); disorders for which disease manifestation could be influenced (7%); UFs providing reproductive options (2%); and UFs with pharmacogenetic implications (5%). Hence, our experience shows that UFs predisposing to medically actionable disorders affect a broader range of genes than listed on the “ACMG59”, advocating that a pre-defined gene list is too restrictive, and that UFs may require ad hoc evaluation of medical actionability. While both the identification and disclosure of UFs depend on local policy, our lessons learned provide general essential insight into the nature and odds of UFs in clinical exome sequencing.

## Introduction

Unsolicited findings (UFs) in clinical genetics are defined as (likely) pathogenic variants not related to the initial clinical question the DNA test was performed for, but that may nonetheless be of medical relevance to the health of the patient and/or his/her family [1] (Box 1).

UFs are variants that are “unsought for”, and have variously been described as “accidental findings”, “co-incidental findings” or “incidental findings”. They differ from “secondary findings” (SFs), which also represent variants not related to the initial clinical question but that are *actively* looked for [2].

Previously, targeted sequence analysis of single genes was performed which made the detection of UFs unlikely. With the implementation of whole exome sequencing (WES) as a first-tier test, analysis is extended to all protein coding genes [3, 4], and consequently, the probability of detecting UFs has increased. This has fostered a worldwide debate on the disclosure of UFs – and SFs – on which consensus has yet to be reached [2, 5].

The American College of Medical Genetics (ACMG) tightened the recommendations for SFs and created a list of 59 so called “medically actionable disease genes” (“ACMG59”) [2]. These genes were selected among the most prevalent monogenic disorders, for which individuals with pathogenic variants remain asymptomatic for a long time, and preventive measures and/or treatment are available [2]. The “ACMG59” list has been widely used, and adopted by others [6–16], within total SFs having been reported in over 100 genes [8, 16] (Box 2).

In contrast to SFs, the recommendations for disclosing UFs have not been updated since 2011 [1]. We hypothesize that differences exist in the prevalence and nature of UFs compared to SFs, but to date this has not been systematically assessed. We present a thorough and systematic analysis of UFs identified during clinical WES of 16,482 index patients and compare the results to SFs from the same clinical population to help establish guidelines for decision making for the disclosure of UFs.

Box 1 Unsolicited findings A medical genetic test is aimed to identify (or exclude) genetic disease underlying a persons’ health condition. With today’s DNA sequencing techniques, an individual’s entire exome or genome can be determined in a single experiment. To identify disease-causing variants, the data are compared to data of healthy controls. These techniques allow the detection of variants that are irrelevant to the clinical question but which predispose to another disease. Such unsolicited findings (UFs) may be of medical value for the patient and family. In this latter context, genetic variants imposing a health risk for blood relatives, such as carrier status of autosomal recessive or X-linked conditions, are considered UFs as well.

Box 2 What is medically actionable? Disclosure of UFs and/or SFs depends on whether an individual receiving the information can medically intervene in the process related to the disorder to which the variant predisposes. The term medical actionability has been criticized for its inexactness [33], leading to multiple interpretations and misinterpretations of health-care-related expectations.

Berg et al. [1] were amongst the first to publish recommendations for the disclosure of both UFs and SFs. They recommended disclosure for variants deemed “medically actionable”, referring to variants carrying a high likelihood of disease (e.g., monogenic, high penetrant disease), and for which medical interventions could significantly reduce morbidity and mortality [34]. Morbidity is defined as “the state of being symptomatic or unhealthy for a disease or condition” and mortality refers to “the number of deaths caused by the health event under investigation” [35]. Berg’s definition has been adopted by the ACMG and others (e.g., Amendola et al. [6] and Dorschner et al. [7]) for the disclosure of SFs. In contrast, less strict definitions include for example the definition used by Yang et al. [8], which states that variants are considered medically actionable when there are potential therapies or established surveillance protocols available.

## Material and methods

### Patient, counseling, and informed consent

Between 01 June 2013 and 31 May 2018, 16,482 consecutive index patients received clinical whole-exome sequencing (WES) in the ISO15189 accredited Genome Diagnostic Laboratory of the Radboud university medical center in Nijmegen, the Netherlands. As part of the counseling and consent procedure prior to performing WES, clinicians informed the patients regarding our two-tiered approach for data interpretation, starting with an analysis of in silico disease gene panels, followed by analysis of the entire exome (see “Two-tiered diagnostic exome sequencing procedure”). Especially the second tier is anticipated to involve the possibility of uncovering an UF. As part of the post-test counseling, patients without a conclusive diagnosis are advised to recontact their referring clinician in due time for re-analysis of the existing exome data as the in silico disease gene panels are revised regularly.

### Two-tiered diagnostic exome sequencing procedure

WES was performed following our routine diagnostic procedures [17] either on the index patient only, or in a family-based trio strategy (index patient + biological parents). From 2015 onwards, also copy number variants (CNV) were routinely identified from WES data and used for diagnostic interpretation [18].

Analysis of WES data were performed as a two-step process, guided by the consent provided by the patient or guardian. The first step, referred to as tier 1, included the analysis of variants restricted to genes known to be associated with the index’s condition by means of an in silico gene panel enrichment (Supplementary Methods). If the patient”s symptoms did not allow for selection of (a) disease-specific gene panel(s), the clinician could also request analysis of the Mendeliome, consisting of all 3606 genes with an OMIM-listed disease-gene association. In case no molecular diagnosis was obtained in tier 1, and the patient consented for further analysis, the analysis was followed by tier 2, allowing for prioritization, interpretation and classification of variants in the Mendeliome (if not already performed in tier 1) and those in genes without known disease-gene associations (“open exome analysis”).

### Variant prioritization, interpretation, and classification

DNA from the index patient and parents was often sequenced simultaneously to facilitate detection and interpretation of de novo variants in autosomal dominant disease genes (Supplementary Methods). Filtering steps and prioritization of variants in the gene panel analysis (tier 1) was performed as described [17]. In tier 2, rare truncating variants and/or known pathogenic variants were assessed. For trios, assessment also included “de novo” and “compound heterozygous” variants (Supplementary Methods). Trio-based analysis allows to determine the inheritance of all variants identified in the index by comparison the variants in the parental samples. It can show that both parents are carrier of the same pathogenic variant that is detected in heterozygous state in the affected child (also a carrier). It does not, however, detect carrier couples which carry different variants in the same recessive risk allele if the child is not compound heterozygous for these variants. A compound heterozygous state will only be uncovered as UF when the child does not present with a matching phenotype. This information is, however, of relevance in the context of unsolicited findings (UF) evaluation, as the couple has a 25% chance of affected offspring in future pregnancies (see “UF disclosure policy” in the Supplementary Methods).

For diagnostic SNV interpretation and classification, we used the guidelines established by the Association for Clinical Genetic Science (ACGS) and the Dutch Society of Clinical Genetic Laboratory Specialists (VKGL). Their 4-class system (UV1–UV4) [19] was used in 2013–2014, and from 2015 onwards, this was exchanged by a 5-class system (Class 1 to Class 5) [20]. CNVs were classified according to the European guidelines for constitutional cytogenomic analysis (Class 1 to Class 5) [21].

### UF evaluation and disclosure policy

During analysis, clinical laboratory geneticists may encounter (likely) pathogenic variants (e.g., UV3/4 or class 4/5), detected in either tier 1 or tier 2, in genes not associated with the disease for which the index was referred. After confirmation of pathogenicity of the variant by a second clinical laboratory geneticist, the variant is subsequently evaluated by an inhouse panel of experts, consisting of a clinical laboratory geneticist, a clinical geneticist, a molecular geneticist, an ethicist, a legal representative and a social worker. The panel assesses whether it is indeed an UF and advises the referring clinician in the disclosure of the UF. Hereto the panel weighs factors such as disease penetrance, severity of disease, the age of onset, the age of the patient, the presumed psycho-social impact, the physical impact of screening program(s) and the time needed to diagnose the genetic disorder without prior knowledge of the UF. The local disclosure policy is provided in the Supplementary Methods.

Of note, this study reflects the first 5 years of clinical WES at our institute. The default consent option was that medically actionable UFs would be disclosed and non-medically actionable UFs would not be disclosed. Medical actionability was interpreted as “the potential to change the course of” or “prevention of” disease by medical interventions in adults or children”, or “when knowledge of the presence of the pathogenic variants allows for early interventions, before or after the first mild symptoms appear”, or “prevention of a diagnostic odyssey”. Carrier status of a recessive disease was also disclosed, provided that the risk to future offspring was at least 25%, as this would allow for reproductive choices.

### Defining UFs eligible for analysis in this study

This study aims to provide the incidence of UFs, observed in index patients receiving clinical WES between 2013 and 2018. To overcome interpretation biases introduced over time due to changes in classification, we have systematically reclassified all UFs in June 2020 using ACMG criteria using information known to date [22]. UFs in eight individuals (5 variants) were excluded because of reclassification from (likely) pathogenic variants (Class 4/5) to a variant of unknown significance (VUS, class 3) and, in two individuals the UF (2 CNVs) was only observed in a parent of the index but not the index him/herself.

Homozygous or compound heterozygous variants in a gene causing a recessive disease were considered a single UF.

## Results

### Odds of UF discovery in diagnostic WES cohort

**Fig. 1:**
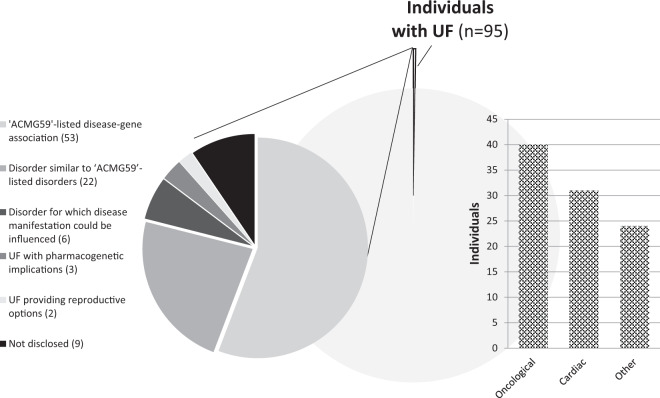
Incidence of UFs in our cohort of 16,482 individuals after clinical exome sequencing and their reasons for disclosure. In 16,482 individuals, UFs were identified in 95 individuals (0.58%). For each gene in which an UF was identified, the medical actionability was evaluated, resulting in six categories (depicted in pie chart on the left). In addition, the disease category to which the UF predisposed was evaluated (depicted in bar chart on the right).

Between 2013 and 2018, a total of 16,482 index patients received WES in our diagnostic laboratory. In total, 97 UFs were identified in 95 patients (two patients had two UFs; Supplementary Table 1). Hence, the odds of detecting an UF in our diagnostic cohort is one in 174 (0.58%; 95/16,482 patients; Fig. 1).

In accordance with our local disclosure policy (Supplementary Methods), the UF was not disclosed to nine of 95 patients because no (national guidelines for) medical intervention would have been applicable (Supplementary Fig. 1). The UF was disclosed to the remaining 86 patients based on the availability of medical interventions. The overall risk of medically actionable UFs in our diagnostic WES cohort is 0.52% (86 of 16,482 patients). For non-medically UFs, it is 0.05% (nine of 16,482 patients).

**Fig. 2:**
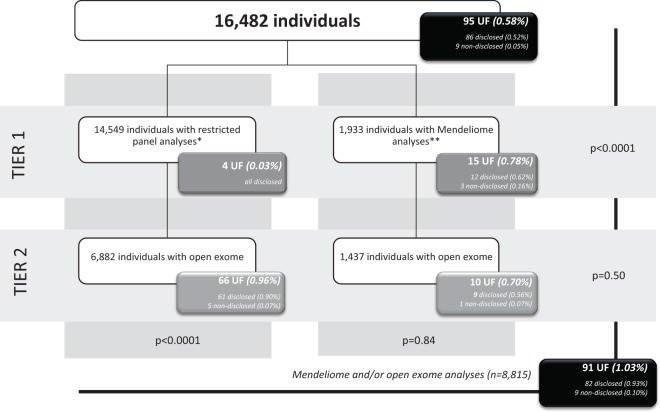
Analysis strategies leading to the disclosure of UFs in 86 of 16,482 individuals after clinical exome sequencing. The odds of UFs after analysis of disease-specific panels were 0.03% (4 of 14,549 individuals), and significantly lower than the odds of UFs in Mendeliome (0.78%; 15 of 1933 individuals; Fisher’s exact, *p* < 0.0001). The odds of UFs in an “open exome” strategy performed after either a targeted disease-gene panel analysis (0.96%), or after the Mendeliome (0.70%), are statistically the same (Fisher’s exact, *p* = 0.45). Similarly, the odds of UFs detected in tier 1 Mendeliome analysis (0.78%) did not significantly differ from the incidence detected in an open exome analysis, regardless whether the tier 1 analysis was a disease-specific panel (0.96% UFs in open exome, Fisher’s exact *p* = 0.50) or a Mendeliome analysis (0.70% UFs in open exome, Fisher’s exact *p* = 0.84). *A panel analysis may consist of the simultaneous interpretation of multiple disease panels, but never includes analysis of the entire Mendeliome; **This analysis consists of at least the Mendeliome, but might include one or more restrictive gene panels.

The UFs in 95 individuals were uncovered via various analysis strategies, each resulting in a different odds of UFs (Fig. 2). For disease-specific panels, this was 0.03% (4 of 14,549 individuals), and for Mendeliome analysis 0.78% (15 of 1933 individuals). The odds of UFs in an “open exome” strategy performed after targeted disease-gene panel analysis were 0.96% (66 of 6882 individuals), and 0.70% after the Mendeliome (10 of 1437 individuals). These results confirm that the probability of uncovering an UF significantly increased when analyzing all genes with proven disease-gene associations (UFs in the Mendeliome and open exome; 91 of 8815 individuals; 1.03%) in comparison to a dedicated disease-gene panel strategy (0.03%; Fishers Exact test *p* < 0.0001).

### Reasons for disclosure of UFs

In 84 of 86 individuals, the UF was disclosed because of a health risk for the index or family, and in two individuals the UF was relevant for reproductive choices of either the index or relatives (Supplementary Fig. 1). Forty-one of the 84 individuals were aged 12 years and over, and the disease the UF predisposed to would be expected to manifest in adolescence or adulthood. In the 43 minors (<12 years of age), 25 UFs were disclosed because the disease has been reported to have a (possible) manifestation in childhood. The other 18 minors were at risk of a disease with adult onset, and the risk was disclosed because of immediate relevance for family members (Supplementary Table 1).

### Comparison to “ACMG59”

The 88 UFs, disclosed to 86 individuals, affected 40 different genes, predominantly predisposing to oncological disease (43%) or cardiac disorders (36%) (Table 1, Supplementary Table 1). Only 20 of these 40 genes (50%) are listed on “ACMG59” [2]. These 20 genes harbor 54 UFs (61%) in 53 individuals. In all but one individual, the UF was identified in the Mendeliome or open exome analysis. The odds of an UF in an “ACMG59”-listed gene was thus 0.59% (52/8815 individuals or one in 170 individuals). The 20 non-“ACMG59”-listed genes harbored 34 UFs in 33 patients. These include 11 genes (22 UFs) associated with diseases that are clinically similar to “ACMG59”-listed conditions, such as predisposition to cancer or cardiac disease. Another group consisted of six genes (one UF each) responsible for diseases for which significant treatment options are available to impact disease manifestation by reducing morbidity. Variants in one gene (four UFs) were disclosed because of the risk of serious adverse drug reactions. UFs in two genes (one UF each) were disclosed because of reproductive choices.

**Table 1:** Levels of medical actionability for the 97 UFs detected in 95 individuals.

PHENOTYPE	“ACMG59” - listed disease-gene association	*Nr*.	“ACMG59”- listed disorder	*Nr*.	Disorder for which disease manifestation can be influenced	*Nr*.	Pharmaco-genetic implications	*Nr*.	Reproductive options	*Nr*.	Not disclosed	*Nr*.
**Oncological**												
Hereditary breast and/or ovarian cancer	*BRCA1*	13	*ATM*	1							*CHEK2*	2
	*BRCA2*	4	*BRIP1*	5								
Lynch syndrome	*MSH6*	2										
	*PMS2*	3										
Familial adenomatous polyposis coli	*APC*	1										
Multiple endocrine neoplasia, type 2	*RET*	2										
Hereditary paraganglioma-pheochromocytoma syndrome	*SDHD*	1	*SDHA*	3								
Melanoma and neural system tumor syndrome			*CDKN2A*	1								
Leiomyomatosis and renal cancer			*FH*	1								
Basal cell nevus syndrome			*SUFU*	1								
**Cardiac**												
Hypertrophic cardiomyopathy, dilated cardiomyopathy	*MYBPC3*	4	*TTN*	2								
	*MYH7*	1	*CSRP3*	1								
	*TNNT2*	1	*FLNC*	1								
	*GLA*	1										
Catecholaminergic polymorphic ventricular tachycardia	*RYR2*	1										
Arrhythmogenic right ventricular cardiomyopathy	*PKP2*	4										
	*DSP*	1										
Romano-Ward long QT syndromes 1, 2 and 3	*KCNQ1*	2^a^										
Brugada syndrome	*KCNH2*	1										
Atrial fibrillation	*SCN5A*	6^a^	*GJA5*	5								
Pulmonal arterial hypertension			*BMPR2*	1								
**Other**												
Familial hypercholesterolemia	*LDLR*	4										
Marfan syndrome	*FBN1*	1										
Malignant hyperthermia susceptibility	*RYR1*	1										
Hailey-Hailey disease					*ATP2C1*	1						
Complement component 8 deficiency type II					*C8B*	1						
Macular corneal dystrophy					*CHST6*	1						
Von Willebrand disease, type 1					*VWF*	1						
Autosomal Recessive Deafness 1A					*GJB2*	1						
Autosomal Recessive Deafness 49					*MARVELD2*	1						
5-fluorouracil toxicity							*DPYD*	4^b^				
Oculocutaneous Albinism									*TYR*	1		
X-linked recessive chondrodysplasia punctata									*ARSL*	1		
Cerebral cavernous malformations											*CCM2*	1
Hypobetalipoproteinemia											*APOB*	1
Frontotemporal dementia, aphasia											*GRN*	2
Muscle glycogenesis											*PHKA1*	1
Autosomal dominant spastic paraplegia Type 12											*RTN2*	1
Legius syndrome											*SPRED1*	1

Details per individual are provided in Supplementary Table 1. *“ACMG59”-listed disease-gene association*: Gene present on the “ACMG59”-list, and UFs predispose to the disease listed; *“ACMG59”-listed disorder*: gene predisposes to a disorder similar to “ACMG59”-listed disorders; *Disorder for which disease manifestation can be influenced*: gene predisposes to a disorder for which disease manifestations can be mitigated; *Pharmacogenetic implications*: UFs with pharmacogenetic implications; *Reproductive options*: UFs with a risk of at least 25% of affected offspring. *Not disclosed*: genes with UFs considered to be not medically actionable.

^a^One individual had one variant in the *KCNQ1* gene and one variant in the *SCN5A* gene.

^b^One individual had two variants in the *DPYD* gene.

## Discussion

In total, we identified UFs in 95 out of 16,842 individuals who received WES, and disclosed UFs in 86 individuals, since we considered them medically actionable. The UFs were uncovered via various analysis strategies, each with a different probability of identifying UFs. From our observations, we learned multiple lessons that provide insights into the nature and odds of UFs in clinical exome sequencing.

### Lesson 1: The incidence of UFs disclosed after clinical exome sequencing is low and depends on variant prioritization and interpretation strategies

Only in four patients, the UF was detected during the analysis of a restricted gene panel, indicating that the likelihood of UF detection in this diagnostic strategy is low (0.03% or 1 in 3637 individuals). In one of the cases, a collodion baby, the UF (in *GJB2*) was uncovered in the gene panel for skin disorders. *GJB2* was included in this panel because dominant negative variants are associated with keratitis-ichthyosis-deafness syndrome. The compound heterozygous loss-of-function variants that were identified in the neonate are associated with a mild form of autosomal recessive deafness type 1. This exemplifies that gene panels may lead to the identification of UFs predisposing to disorders outside the expertise of the WES requesting physician. In the three other cases, the UF predisposed to a different disease within the disease spectrum analyzed. In an 18-year-old man, the UF in *GLA* predisposed to a later onset disease. In two other index patients, phasing of variants revealed at least 25% risk for their parents of having affected offspring: a heterozygous *ARSL* (X-linked) variant in a female index patient was maternally inherited, and a heterozygous *TYR* variant identified in a 5-year-old girl, was also present in both her parents. These examples highlight the importance of awareness of the gene panel contents to enable adequate counseling of the probability of UFs.

The probability of uncovering an UF in the Mendeliome and/or open exome was significantly higher (1.03% or one in 97 individuals) than in a disease-gene panel, suggesting that the risk of uncovering an UF is related to the number of known disease genes analyzed, as has been postulated before [23]. With these odds, one may question whether the probability of detecting UFs exceeds the chance of finding the genetic cause of disease after a negative restricted gene panel analysis. The answer to this question cannot be given unequivocally as this is largely determined by the extent to which the clinical heterogeneity of the primary condition is already captured with laboratory-specific disease gene panels, and will vary between diseases and clinical laboratories.

### Lesson 2: UFs can occur during re-analysis of existing data

Patients without a genetic diagnosis are often advised to recontact the clinician for reanalysis of their existing exome data because of increasing knowledge on genes and variants involved in disease, the implementation of new bioinformatic pipelines, and novel sequence technologies. Together this may allow detection of the disease-causing variant (several) years after the initial analysis. The same is true for uncovering UFs: six UFs were identified and disclosed after a request for clinical reanalysis of such existing exome data, performed two to five years after the initial WES analysis. These findings underscore the importance for clinicians to address the possibility of identification and disclosure of so far unidentified UFs, before requesting re-analysis. Moreover, it confirms the notion that not all medically actionable disease-gene variants will be seen upon testing if not actively looked for. Hence, when no UFs are disclosed after clinical exome sequencing, patients should not falsely deduce absence of genetic predisposition for medically actionable diseases.

### Lesson 3: The odds of UFs in “ACMG59” are substantially lower than for SFs

The odds of UF discovery depend on variant prioritization and interpretation strategies used in the clinical laboratory. Similarly, the incidence of SFs reported varies because of differences in inclusion criteria, ethnicity, sequencing techniques, and variant interpretation criteria [6–16], which limits the comparison of results between studies. To take away these biases, we compared the data on UFs from this study to our published data on SFs from the same population, for which we reported an incidence of 1:38 individuals (2.6%) for medically actionable dominant diseases listed on the “ACMG59” gene list [2, 24].

In 54 individuals UFs in ACMG-listed genes were identified (Supplementary Table 1). One was not disclosed because the variant did not predispose to the ACMG-listed disease (*APOB*) and another UF was identified through panel-based analysis. We thus identified 52 UFs in 8815 individuals (0.59%) receiving Mendeliome/open exome analysis in genes listed on the “ACMG59”. This results in an odds of 1:170, which is fourfold lower than the incidence observed for SFs (1:38) [24]. This difference reflects our variant prioritization strategies since variants need to be clearly recognizable as pathogenic in order to be noticed. Truncating, or other loss-of-function variants, are more likely to be noticed than missense variants, because of their more obvious impact on protein function. Hence, variants for diseases caused by haploinsufficiency will be more easily recognized, even if the exact variant has never been reported before. For (rare) missense variants, pathogenicity is less obvious, requiring more extensive analyses. Indeed, we observed more loss-of-function UFs in “ACMG59”-listed genes (57%), than we did for SFs in the same genes (35%) [24]. Also, trio-based filter strategies are biased away from inherited autosomal dominant disease-causing variants (i.e., the vast majority of “ACMG59”-listed disorders), as diagnostic prioritization is focused towards de novo and recessive variants. The four-fold difference between the odds of UFs (1:170) and incidence of SFs (1:38) that we observed, can however not be generalized to other clinical laboratory programs, as it depends on multiple factors, including local variant prioritization strategies. Nonetheless, we expect that other clinical laboratories will also observe a lower odds for UFs than SFs as they prioritize disease-causing variants related to the clinical question.

### Lesson 4: Medical actionability for UFs differs from “ACMG59” recommendations

Only 54 of 88 disclosed UFs (61%) involved an “ACMG59”-listed gene. Medical actionability of the diseases listed is based on prevention and reduction of mortality and morbidity. The remaining 34 UFs (39%) were identified in twenty genes not listed on the “ACMG59’. The reported contribution of non-ACMG genes to the incidence of SFs ranges from 13 to 52% [6–11, 16]. Eleven genes we report, however, predispose to the same conditions as listed on “ACMG59”, such as breast cancer (*BRIP1*, five UFs) and cardiac disease (*CSRP3*, one UF), or predispose to conditions that fall in the same phenotypic spectrum of diseases, such as renal cancer (*FH*, one UF) and pulmonary hypertension (*BMPR2*, one UF). The low prevalence of these genes in causing these disorders may be a reason why “ACMG59” has not included this well-known extensive genetic heterogeneity [2]. Additionally, using a fixed list means that population-specific founder variants may not be taken into account as exemplified in our study by a recurrent and relatively prevalent Dutch founder variant in *SDHA* (three UFs) [25]. These findings show the limited applicability of “prevalence” as a universal criterion for disclosing UFs. A proposal to expand the “ACMG59” to over 100 genes to overcome the genetic and clinical heterogeneity of the listed disorders has been made before [8, 16].

We disclosed six other variants that may allow individuals to undergo medical interventions, aiming at influencing the course of disease rather than preventing it. For instance, *GJB2* (one UF) and *MARVELD2* (one UF) cause early-onset hearing loss, which itself cannot be prevented, but morbidity associated with the hearing loss, such as speech and language delay, can be mitigated at a young age. Similarly, an UF in *VWF* was disclosed because of potential for intervention with medication to optimize and maintain hemostatic stability. As these interventions reduce morbidity, these UFs fulfill the criteria of being medically actionable and thus represent a category of diseases for which UF disclosure could be considered.

### Lesson 5: The odds of UFs depend on (local) disclosure policy

The most prominent reason for disclosure of UFs in our policy has been their medical actionability. The expert panel also discussed nine UFs predisposing to disorders for which medical interventions to reduce mortality or morbidity did not apply. After review, these were not disclosed to the family. One example includes frontotemporal dementia (*GRN*, two UFs), for which worldwide, no medical management is available. We also did not disclose variants for *CHEK2*-associated susceptibility to breast cancer (two UFs) because no national screening programs have been established for this condition in absence of familial breast cancer [26]. Interestingly, we noted that after an initial policy decision not to disclose *CHEK2* variants, our laboratory geneticists refrained from further reporting variants in this gene to the expert panel.

Similar low odds are observed for variants facilitating reproductive and pharmacogenetic options. In total two UFs, in two different genes (*TYR-* autosomal recessive inheritance, and *ARSL*-X-linked inheritance) were disclosed because of the health risk for future offspring. In both cases, parents of the index had at least 25% of having an affected child with a disorder manifesting at birth or early childhood. Only one couple was at risk of an autosomal recessive disorder, which is far less than the empirical ~1% which could have been identified [27]. Parents did not receive WES themselves for the purpose of carrier analysis. Our approach only allowed the detection of couples carrying the same pathogenic variant (thus at risk of a homozygous child). Couples at risk of a compound heterozygous child are not detected in our study, whereas they are included in the empirical 1% of couples at risk of an autosomal recessive disease. Notably, should we not have used the threshold of ≥25% risk of affected offspring, the odds of UFs in this category would have significantly increased since it is estimated that every individual is a carrier of at least two pathogenic variants in currently known autosomal recessive genes [27]. We also disclosed four UFs of pharmacogenetic relevance, all identified in *DPYD*, which is low given that many more genes are known to be of importance for management of the optimal dose of medication [28]. Most of the variants in these genes are common variants requiring special expertise to recognize these UFs. This assumption was confirmed by the observation that the UFs in *DPYD* were identified by clinical laboratory geneticists with specific expertise in pharmacogenomics. Hence, the odds of UFs in pharmacogenetic genes are not representative of the incidence of pharmacogenetic relevant variants in clinical WES. For both reproductive options and pharmacogenetics, dedicated genetic tests can be performed to assess individual risk of a specific situation such as preconception carrier testing or pharmacogenetic passport.

The default option of our local policy was to only disclosure medically actionable findings. All patients consented as alternative (targeted) diagnostic testing opportunities were offered. With WES becoming a first-tier diagnostic test, offering opt-out options for medically actionable disease and opt-in for non-medically actionable disease has become a matter of intense debate. As a result, opt-out/opt-in options have been implemented in a Dutch national consensus-based guidance in 2021 (https://vkgl.nl/nl/diagnostiek/incidental-findings). The extent to which these options will affect UF disclosure remains to be seen and will allow to register how many patients will choose to “opt-out” from hearing UFs, or opt-in for non-medically actionable disease.

With the ongoing debate on disclosing UFs, we believe that our evaluation of UFs observed in everyday diagnostic practice collected over a five-year period on >15,000 exomes, provides valuable perspectives on the clinical impact and utility of UFs. Concerns have been raised about the penetrance of genetic variants in the context of UFs/SFs which has led genomic professionals to question their utility [29–31]. It would be of great value to describe the follow-up of patients to whom an UF was disclosed to evaluate their clinical relevance. For a subset of 20 individuals with appropriate consent for recontact, we have performed qualitative interviews regarding their experiences and preventative measures they have taken [32]. Only a minority of our participants experienced symptoms related to the UF. However, it has been beyond the scope of the current manuscript to follow-up on the medical relevance of all of these UFs.

## Conclusion

The odds of UFs in our diagnostic workflow are low, ranging from 0.03 to 1.03% for analysis of disease-gene panels and the entire exome, respectively. Our local disclosure policy had a large impact. Our observations that UFs, defined by *ad-*hoc review of medical actionability, affected a broader range of genes than listed on “ACMG59”, suggest that pre-defined gene lists may need to be reconsidered.

## Supplementary information

Supplementary Methods

Supplementary Figure 1

Supplementary Table S1

## Data Availability

All data generated or analyzed during this study regarding UFs are included in this published article and its supplementary information files. Individual exome sequencing data of all 16,482 indexes cannot be disclosed.
