# Supplementary material for: Lessons learned from unsolicited findings in clinical exome sequencing of 16,482 individuals
Source: Eur J Hum Genet. 2021 Oct 25;30(2):170–7. doi: 10.1038/s41431-021-00964-0 (PMC8821629; doi:10.1038/s41431-021-00964-0)
Supplement: Supplementary file 1 — Supplementary Methods [file 41431_2021_964_MOESM1_ESM.docx]

**Supplement to**

Lessons learned from unsolicited findings in clinical exome sequencing of 16,482 individuals

Vyne van der Schoot MD^1^*, Lonneke Haer-Wigman PhD^2,3^*, Ilse Feenstra MD, PhD^2^, Femke Tammer MD^2^ , Anke JM Oerlemans PhD^4^ , Martine PA van Koolwijk^2^, Frans van Agt^5^, Yvonne HJM Arens MD, PhD^1^,

Han G Brunner MD, PhD^1,2,6^, Lisenka ELM Vissers PhD^2,3, ¥^ and Helger G Yntema PhD^2,3, ¥^

^1^Department of Clinical Genetics, Maastricht University Medical Center, PO Box 5800, 6202 AZ Maastricht, The Netherlands; ^2^Department of Human Genetics, Radboud university medical center, PO Box 9101, 6500 HB Nijmegen, The Netherlands; ^3^Donders Institute for Brain, Cognition and Behaviour, Radboud university medical center, Nijmegen, the Netherlands ^4^IQ healthcare, Radboud Institute for Health Sciences, Radboud university medical center, PO Box 9101, 6500 HB Nijmegen, the Netherlands; ^5^Commissie Mensgebonden Onderzoek, Research Ethics Committee, PO Box 9101, Internal Code 578, 6500 HB, Nijmegen, The Netherlands; ^6^Radboud Institute for Molecular Life Sciences, Radboud University, Nijmegen, the Netherlands.

*These authors contributed equally

¥These authors jointly supervised the work

Correspondence to:

Helger Yntema, PhD

Radboud university medical center

Department of Human Genetics

P.O. box 9101

6500 HB Nijmegen

[helger.ijntema@radboudumc.nl](about:blank)

T +31-24-3613799/ F +31-24-3616658

**Supplementary Methods**

**Technical details of Whole Exome Sequencing procedure**

WES was performed following our routine diagnostic procedures(1) either on the index patient only, or in a family-based trio strategy (index patient + biological parents). In brief, DNA was outsourced to BGI Copenhagen (Denmark) for WES using an Agilent v4 (June 2013 - March 2015) or v5 (April 2015 - June 2018) all Human Exon Enrichment kit, followed by sequencing on HiSeq2000 or HiSeq4000 sequencer (Illumina) to a median sequence depth of at least 75-fold. FASTQ files were subsequently provided to our laboratory, and run through a custom diagnostic bioinformatic pipeline for variant calling (GATK) of single nucleotide variants (SNV) and insertion deletion events. For each variant, annotation allowing prioritization was added, including, amongst others, variant effect prediction, population frequencies and previous reports of pathogenicity. From 2015 onwards, also copy number variants (CNV) were routinely identified from WES data, annotated and used for diagnostic interpretation(2).

**Analysis of data**

Analysis of WES data was performed in a two-step process, guided by the consent provided by the patient. In the first step, referred to as tier 1, the referring clinician selected the most appropriate *in silico* disease gene panel(s), listing between 56 and 1,159 genes per panel. If the patient’s symptoms did not allow for selection of (a) disease-specific gene panel(s), the clinician could also request analysis of the Mendeliome, consisting of all 3,606 genes with an OMIM-listed disease-gene association. In case no molecular diagnosis was obtained in tier 1, and the patient consented for further analysis, the analysis was followed by tier 2. An overview of the number of genes per panel, requested in patients in whom an IF was identified, is listed in the table below:

|  |  | Number of genes in panel *(v.DG-2.14.0)* | |
| --- | --- | --- | --- |
| *Restricted gene panel* | | *Total* | *of which ‘ACMG59’ listed genes* |
| *Hereditary cancer* | | 206 | 25 |
| *Skin disorders* | | 611 | 19 |
| *Intellectual disability* | | 1,159 | 7 |
| *Metabolic disorders* | | 625 | 5 |
| *Muscle disorders* | | 157 | 4 |
| *Renal disorders* | | 252 | 4 |
| *Epilepsy* | | 316 | 2 |
| *Movement disorders* | | 304 | 2 |
| *Haemostatic/thrombotic disorders* | | 145 | 1 |
| *Ciliopathies* | | 146 | 1 |
| *Disorders of sex development* | | 56 | 1 |
| *Hearing impairment* | | 168 | 1 |
| *Neuropathies* | | 96 | 1 |
| *Primary immunodeficiencies* | | 386 | 0 |
| *Vision disorders* | | 415 | 0 |
| *Mendelian inherited disorders* | | 3,606 | 59 |

Of note, the number of genes as listed is based on the panel content of June 2018 (version DG-2-14.0). A current overview of all (previous) panel releases is presented on-line (<https://www.radboudumc.nl/en/patientenzorg/onderzoeken/exome-sequencing-diagnostics/information-for-referrers/exome-panels>)

**Variant prioritization and interpretation for trio-based analysis**

Index patients were frequently sequenced simultaneously with their parental samples (‘trio-analysis’) to allow identification and interpretation of *de novo* variants in autosomal dominant disease genes. That is, trio-based analysis allows to determine the inheritance of all variants identified in the index by comparison the variants in the parental samples. For SNVs, this strategy precludes the identification of variants solely identified in (one of) the parent(s). It can show for instance that both parents are carrier of the same pathogenic variant that is detected in heterozygous state in the affected child (also a carrier). This information is however of relevance in the context of unsolicited findings (UF) evaluation, as the couple does have a 25% chance of affected offspring in future pregnancies (see ‘UF disclosure policy’).

For CNVs, analysis is complicated by fragmentation of called segments, and requires additional graphical representation of the data in a genome-wide view to establish inheritance of the variants and interpretation. The latter may thus lead to the visual observation of CNVs only identified in (one of) the parent(s), but which is absent in the index. Whereas these CNVs can be considered an UF for disclosure to the parent(s) if it for instance has clinical implications (see ‘UF disclosure policy’), we have excluded these UFs from our analysis here to provide an overview of UFs identified in the index cases receiving clinical exome sequencing.

**UF disclosure policy *(applicable to clinical WES between 2013-2020)***

The policy of the Department of Human Genetics of the Radboudumc applies to UFs, as there is NO active search for disease causing variants in genes that have no relation to the disease for which the patient is referred to by the treating physician. This policy was based on published European points of consideration for the disclosure of UFs(3).

*General remarks*

A genetic variant for which there is insufficient proof of pathogenicity, is not considered to be an UF. For the 4-class system(4), used until 2015, this refers to variants classified as UV1 and UV2. For the 5-class system(5) of SNVs and indels, used 2015 onwards, this refers to Class 1, Class 2 and Class 3 variants. For CNVs, this refers to Class 1, Class 2 and Class 3 CNVs according to the European guidelines for constitutional cytogenomic analysis(6).

UFs will only be reported during an ongoing clinical consultation. In the event that a variant is reclassified based on novel knowledge gained, it is considered good clinical practice to recontact the patient and send a revised report for variants disclosed as UFs that were wrongly deemed (likely) pathogenic.

*Variants with a potential health risk for the patient (or his blood relatives)*

In principle, UFs that, at time of discovery, cause a disease which course CANNOT be changed by medical intervention, will NOT be reported.

Mentally competent individuals aged 12 and above will be informed on UFs relevant for their own health (or for that of their blood relatives) when medical intervention is possible.

For minors below the age of 12, UFs related to a childhood-onset disease (manifestation under the age of 16) for which medical intervention is possible will ALWAYS be disclosed.

For minors below the age of 12, UFs increasing the risk of adult-onset diseases WILL NOT be disclosed. Nonetheless, UFs of potential medical relevance to one of the parents WILL BE disclosed if options to medically intervene are available.

*Variants with a potential health risk for the patient’s unborn progeny (or for the unborn progeny of his blood relatives)*

UFs related to genetic carrier status, will - in principle - NOT be disclosed as they, by definition, are NOT of medical relevance to the patient himself. Nonetheless, carrier status exposing the carrier, or couple, at a risk of at least 25% of conceiving a child with a genetic disorder WILL BE disclosed.

**References to Supplementary Methods**

1. Haer-Wigman L, van Zelst-Stams WA, Pfundt R, van den Born LI, Klaver CC, Verheij JB, et al. Diagnostic exome sequencing in 266 Dutch patients with visual impairment. Eur J Hum Genet. 2017;25(5):591-9.

2. Pfundt R, Del Rosario M, Vissers L, Kwint MP, Janssen IM, de Leeuw N, et al. Detection of clinically relevant copy-number variants by exome sequencing in a large cohort of genetic disorders. Genet Med. 2017;19(6):667-75.

3. Vears DF, Senecal K, Clarke AJ, Jackson L, Laberge AM, Lovrecic L, et al. Points to consider for laboratories reporting results from diagnostic genomic sequencing. Eur J Hum Genet. 2018;26(1):36-43.

4. Bell J, Bodmer D, Sistermans E, Ramsden S. Practice guidelines for the Interpretation and Reporting of Unclassified Variants (UVs) in Clinical Molecular Genetics. Clin Mol Genet Soc. 2006.

5. Wallis Y, Payne S, McAnulty C, Bodmer D, Sistermans E, Robertson K, et al. Practice guidelines for the evaluation of pathogenicity and the reporting of sequence variants in clinical molecular genetics. Association for Clinical Genetic Science and the Dutch Society of Clinical Genetic Laboratory Specialists. 2013.

6. Silva M, de Leeuw N, Mann K, Schuring-Blom H, Morgan S, Giardino D, et al. European guidelines for constitutional cytogenomic analysis. Eur J Hum Genet. 2019;27(1):1-16.
