# Supplementary material for: Lessons learned from unsolicited findings in clinical exome sequencing of 16,482 individuals
Source: Eur J Hum Genet. 2021 Oct 25;30(2):170–7. doi: 10.1038/s41431-021-00964-0 (PMC8821629; doi:10.1038/s41431-021-00964-0)
Supplement: Supplementary file 2 — Supplementary Figure 1 [file 41431_2021_964_MOESM2_ESM.docx]

**Supplementary Figure 1. Dissemination of UFs in 95 individuals**

***Supplementary Figure 1*** Flow diagram representing the results of the evaluation process for the disclosure of UFs as guided by our inhouse policy for UF disclosure. Numbers listed in the boxes represent the number of individuals. *: index or parent(s) is are carrier of an X-linked or autosomal recessive condition.
